# Effect of Scanning and Support Strategies on Relative Density of SLM-ed H13 Steel in Relation to Specimen Size

**DOI:** 10.3390/ma12020239

**Published:** 2019-01-11

**Authors:** Tomasz Kurzynowski, Wojciech Stopyra, Konrad Gruber, Grzegorz Ziółkowski, Bogumiła Kuźnicka, Edward Chlebus

**Affiliations:** Faculty of Mechanical Engineering, Centre for Advanced Manufacturing Technologies (CAMT/FPC), Wroclaw University of Science and Technology, 50-371 Wrocław, Poland; tomasz.kurzynowski@pwr.edu.pl (T.K.); konrad.gruber@pwr.edu.pl (K.G.); grzegorz.ziolkowski@pwr.edu.pl (G.Z.); bogumila.kuznicka@pwr.edu.pl (B.K.); edward.chlebus@pwr.edu.pl (E.C.)

**Keywords:** selective laser melting, H13 tool steel, process parameters, scanning strategy, support strategy, porosity reduction

## Abstract

Standard experimental research works are aimed at optimization of Selective Laser Melting (SLM) parameters in order to produce material with relative density over 99% and possibly the highest scanning speed. Typically, cuboidal specimens with arbitrarily selected dimensions are built. An optimum set of parameters, determined on such specimens, is used for building parts with variable cross-section areas. However, it gives no guarantee that the density of variable-section parts produced with so selected parameters will be as high as that of the specimens measured during the parameters optimization process. The goal of this work was to improve the process of SLM parameter selection according to the criterion of maximum relative density, based on the example of AISI H13 tool steel (1.2344). A selection method of scanning strategy ensuring relative density of parts over 99%, irrespective of their dimensions, was determined. The specimens were produced using several variants of support structures. It was found that proper selection of the support strategy prevents development of columnar pores.

## 1. Introduction

The SLM technology belongs to methods of additive manufacturing, where a part is built by adding subsequent portions of material in the form of a thin powder layer that is then selectively melted by a laser beam. This technology can be successfully used in aircraft (fuel injection nozzles), automotive (thermal screens), tooling (injection moulds) and medical (individualized implants) industries [1,2].

By SLM technology, it is possible to manufacture parts of materials like nickel-based superalloys and alloys based on titanium, cobalt, aluminum and iron [3]. Among iron alloys, stainless steels like 316L (1.4404), 15-5PH (1.4540) and 17-4PH (1.4542) are commercially applied for manufacture of parts by SLM. A material commonly used in this method is tool steel H13 (1.2344), applied for injection and casting moulds, worms and cylinders for processing of plastics and tools for extrusion of light-metal profiles. This steel is characterized by high tensile strength, hardness and abrasion resistance at high temperatures. Application of SLM to manufacture of injection moulds of H13 steel gives great possibilities for designing complex geometries including conformal ducts. It should be noted that this complexity means high variability of the cross-section area of the mould along its built direction, which should be considered already at the stage of developing the process window.

By this technology, it is possible to build both thin-walled parts with minimum dimension equal to laser spot diameter (80–200 µm) and solid parts with outside dimensions limited by the size of the working chamber. However, manufacture of high-quality parts with a strongly variable cross-sectional area can cause problems with selection of process parameters precluding occurrence of defects like pores, cracks and deformations.

Cracks and geometry distortions of parts result from creation, during rapid solidification of thin layers of molten metal, significant thermal gradients generating residual stresses. Some attempts are made to reduce these stresses, such as double scanning of each layer, scanning of small areas (chessboard type), application of support structures or heating-up the working platform [4].

In the so-far performed research, attention was mostly paid to defects in the form of pores differing in shape, size and formation mechanism [5,6], with respect to their negative influence on ductility and fatigue strength of parts. The shape of pores (spherical, spheroidal, key-hole, irregular, elongated) depends on their formation mechanism, i.e., due to trapping gases by surface turbulences of the liquid pool and local sinking of the seam, to incomplete melting of powder and to shrinkage [7,8].

Spherical pores (gas pores) are formed during melting powder with increased humidity due to much lower solubility of gases in solid solution in comparison to liquid metal. This is especially visible during processing powders of aluminum alloys [9]. Gas pores can also result from entrapped gas in powder particles and from the Marangoni effect, described as the mass transfer along an interface between two fluids due to a gradient of the surface tension [10].

Key-holes are usually created during the process, when the ratio of the liquid pool depth to half of its width is larger than 1 [11,12]. Then, a tunnel filled with evaporating metal and shield gas is formed in front of the pool face. When the rapidly solidifying metal prevents gases from getting out from under the pool, gas pore type key-holes are created.

Elongated interlayer pores (lack of fusion) occur at the boundary between subsequent travels of a laser beam. The formation of this type of pore is related to too short a lifetime of the liquid metal pool, its wettability, improper selection of the distance between scanning lines or too high scanning speeds. During melting, quickly solidifying liquid metal is unable to fill free spaces, creating later interlayer pores [7,13]. Recent studies and in-process monitoring methods allow prediction of a lack of fusion based on melt pool monitoring data [14].

Columnar pores originate under the conditions of too low energy density, when small discontinuities of the melted layer can develop into defects with their shape similar to tunnels running through many layers [15].

On the grounds of the hitherto carried-out examinations, it was found that the tendency of pore formation in parts manufactured by SLM depends strongly on process parameters, specifically laser power, scanning speed and scanning strategy [5]. Thus, numerous studies focused on investigation of the influence of these parameters on the type, number and arrangement of pores in order to know their formation mechanisms. As was already stated by Gustmann et al. [16] for Cu-Al-Ni-Mn, scanning strategy plays an important role in affecting the quantity of pores, their size and arrangement. Arrangement of pores is dependent on the points characteristic for the given strategy and direction of the laser beam movement. For example, for the strategy “scanning strips in the same direction”, Gustmann et al. [16] stated that pores occur along the scanning line and the areas scanned at the beginning show a smaller numbers of pores. In the contour strategy, however, pores are generally arranged in return points of the scanning line, and for the chequered patter, pores occur at the boundaries between fields and at some distance from the scanning initial point. For the considered H13 steel, Beal et al. [17] found a strong influence of the scanning strategy on relative density depending on the applied distances between the lines (hatch distance).

In spite of numerous studies carried-out in this field, the relationships between scanning strategy and the mechanism of formation of pores, especially columnar ones, remain unclear. In addition, there is no research concerning the influence of the cross-sectional area of the built part on relative density. This is significant from the viewpoint of parts with complex shapes, whose manufacture by SLM can be possibly competitive against the traditional technologies.

Support structures are used in laser additive manufacturing to support the parts being built and work as a heat conductor removing heat used in the process, which makes the internal thermal conditions stable during the process. Gan and Wong [18] showed that orientation and distribution of support structures influence levelness of built parts. It seems that a support strategy properly selected to the scanning strategy can significantly affect the creation of pores in the first solidified layers. 

The presented work was aimed at determining, based on the example of H13 steel, the influence of the scanning strategy on the amount, shape and arrangement of pores in manufactured specimens depending on their dimensions. The specimens were produced with the additional use of several variants of supporting structures and their inclination angle to the scanning direction, in order to ensure uniform heat dissipation and to prevent columnar pore formation.

## 2. Materials and Methods 

**Figure 1:**
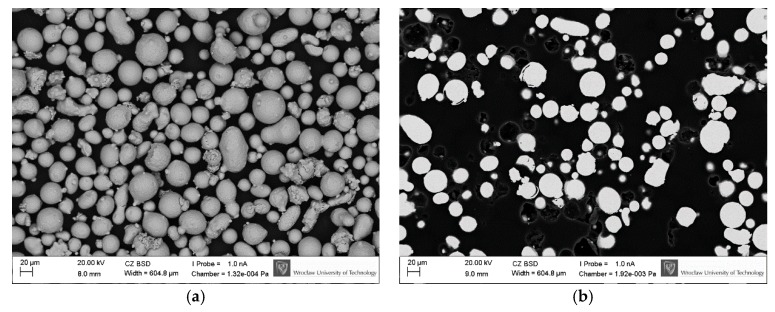
Quality of steel H13 powder: (**a**) spherical and spheroidal shapes, particles surfaces with small amount of satellites; (**b**) cross-sections of particles free from gas pores.

Specimens were made of H13 steel powder, sieved through a screen with a mesh size 63 µm. Next, powder was subjected to chemical analysis, evaluation of particle shapes by Scanning Electron Microscope (SEM, EVO MA25, ZEISS, Oberkochen, Germany) observations and measurement of particle sizes by image computer analysis with use of MicroMeter software (Version 1.0, Warsaw University of Technology, Warsaw, Poland). SEM observations showed spherical or spheroidal shapes of powder particles (Figure 1a) and the absence of closed pores (Figure 1b).

**Figure 2:**
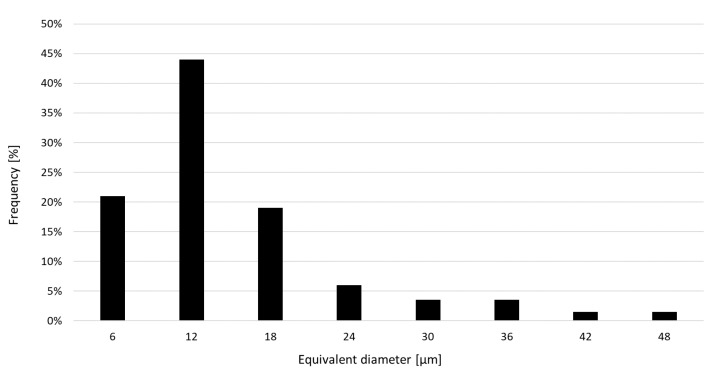
Distribution of fractions of H13 powder used in the research.

Image analysis of cross-sections of particles (Figure 1b) showed that maximum diameters of particles did not exceed 50 µm (Figure 2). The highest percentage of 45% was found for particles with equivalent diameter of 12 µm. Percentages of particles with equivalent diameters 6 µm and 18 µm were about 20% each. Such distribution of fractions corresponded to the bulk density of powder at the level of 4.6 g/cm^3^, i.e., 59% density of solid material (7.8 g/cm^3^). As can be seen in Figure 2, the used powder is fine and the distribution of its particles size is favorably skewed towards smaller diameters. The powder with a larger portion of fine particles provides a higher density of the bed, higher density of created parts under low laser energy and generates smoother side surfaces of finished parts [19].

The chemical composition of the powder is given in Table 1. Before processing, the powder was soaked at 200 °C for 48 h in order to reduce its humidity thus increasing flowability, and to reduce its tendency to be covered with an oxide layer and, in consequence, to increase its processability. 

Specimens were manufactured using an SLM Realizer II 250 (Realizer, Borchen, Germany) machine equipped with a Yb fibre laser with maximum power of 400 W, wave length of 1070 nm and focused laser beam diameter of 200 µm. Material was processed under an argon shield without heating the working platform. All the specimens were manufactured with the use of the optimum set of parameters, given in Table 2. That set of parameters was determined by manufacture, using alternative scanning strategy, of specimens 8 mm × 10 mm × 5 mm (x × y × z) with minimum relative density 99.8 % as the acceptance criterion. 

Tests were carried out in three stages in that effect of selected factors on relative density of the manufactured specimens was determined, namely:

- stage I—effect of scanning strategy depending on cross-section (x × y) dimensions of specimens (4 mm × 5 mm; 8 mm × 10 mm and 16 mm × 20 mm ) and height of 5 mm (z),
- stage II—effect of support structures,
- stage III—effect of intermediate layers (connecting supports with standard layers) built with lower laser power.

**Figure 3:**
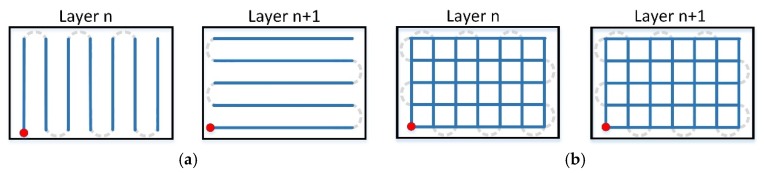
Standard scanning variants: (**a**) single scanning—A0; (**b**) double scanning—B0.

**Figure 4:**
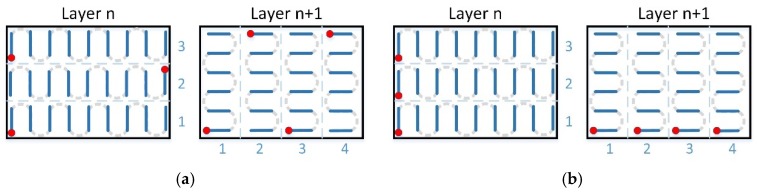
Standard single scanning variants: (**a**) stripes alternating—A1; (**b**) stripes in the same direction—A2.

**Figure 5:**
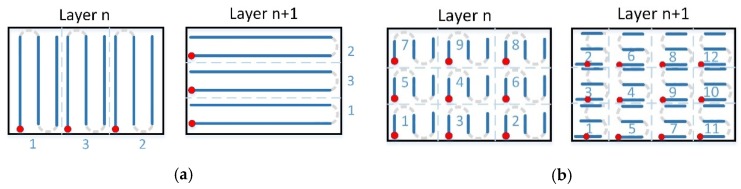
Standard single scanning variants: (**a**) stripes alongside—A3; (**b**) chessboard—A4.

At stage I, specimens with various dimensions were manufactured using 2 scanning variants for each size: single (A) and double (B) (re-melting), and 4 strategies (apart from standard strategies with long scan vectors (A0 and B0, see Figure 3), without dividing the scan area into sections) for each of the variants (A) and (B), see Figure 3:

1. Strategy (1) consisted of alternating scanning of stripes, where subsequent stripes are scanned from the edge of that scanning after the previous strip was finished (Figure 4a).
2. Strategy (2) consisted of scanning strips in the same direction, where starting points of scanning individual stripes were located on the same edge (Figure 4b).
3. Strategy (3) consisted of longitudinal scanning of stripes, similar to standard scanning, while scanning of individual stripes took place in a strictly determined order (Figure 5a).
4. Strategy (4), so-called chessboard, consisted of scanning individual square fields relative to those for which the specimen surface was divided. The scanning sequence of the fields is marked in Figure 5b.

Patterns of scanning Strategies 1–4 are shown in Figure 4 and Figure 5 for the variant of single scanning (A). In the variant (B) of double scanning of each layer, Strategies 1–4 are performed in a similar way as strategy 0 (see Figure 3b). The scanning pattern of the solidified and again melted ”layer n” is the same as that of ”layer n+1” of the single scanning variant (A) (see Figure 4 and Figure 5). This means that the scanning, i.e., powder melting and re-melting with no powder addition, is identical for each layer.

In strategies 1, 2 and 3, various widths of scanning stripes were applied (e.g., A1/2—2 mm, A1/3—3 mm and A1/8—8 mm), but in the chessboard strategy—various sizes of individual fields were applied (e.g., A4/2—2 mm × 2 mm, A4/3—3 mm × 3 mm, A4/8—8 mm × 8 mm).

At stage II, specimens were manufactured using the strategies A3/3 and A4/3, and new supporting structures were designed. The supports used at stage I were located 2 mm away from each other and coincided with the scanning axes X and Y. At the second stage, the distances were reduced to 1.5 mm and the supports were inclined to the axis X at 30° and 45°.

At stage III, two series of specimens were manufactured with use of laser power reduced from 200 W to 150 W and with support structures located 1.5 mm away from each other, inclined to the axis X at 5°, 10° and 30°. In the second series, unlike in the first one, the first 5 layers from the support side were produced at laser power 125 W and the subsequent layers at 200 W. These intermediate layers served as a heat sink for the subsequent standard layers.

Relative density of the specimens manufactured at all the stages was assessed by computer image analysis. Metallographic polished sections were prepared on the planes xy. Surface images obtained with the use of a confocal microscope were subjected to binarization. Porosity was determined as the percentage of pixels falling on pores, i.e., on each kind of empty area irrespective of its shape and origin. Thus, ”relative density” in this paper is calculated as the difference between 100% and percentage of pores.

Detection and visualization of three-dimensional distribution of pores within the specimens 16 mm × 20 mm was carried-out by computed tomography (CT, METROTOM 1500, ZEISS, Oberkochen, Germany). Reconstruction was performed using the system ZEISS METROTOM 1500 (Oberkochen, Germany). Resolution of data for steel specimens was 29.81 µm, at the X-ray tube voltage 225 kV and current 130 µA, with integration time of a single projection at the level of 2 s. With regard to the high density of the reconstructed specimens, a copper filter 3 mm thick was applied, and the obtained reconstructions were corrected to minimize measurement artefacts in the form of beam hardening. For the obtained data, porosity was detected with use of the software VG Studio MAX 2.0 (Volume Graphix, Heidelberg, Germany) and relative density was calculated as described above.

## 3. Results

### 3.1. Effect of Scanning Strategy on Relative Denisty Depending on Specimen Dimensions

**Figure 6:**
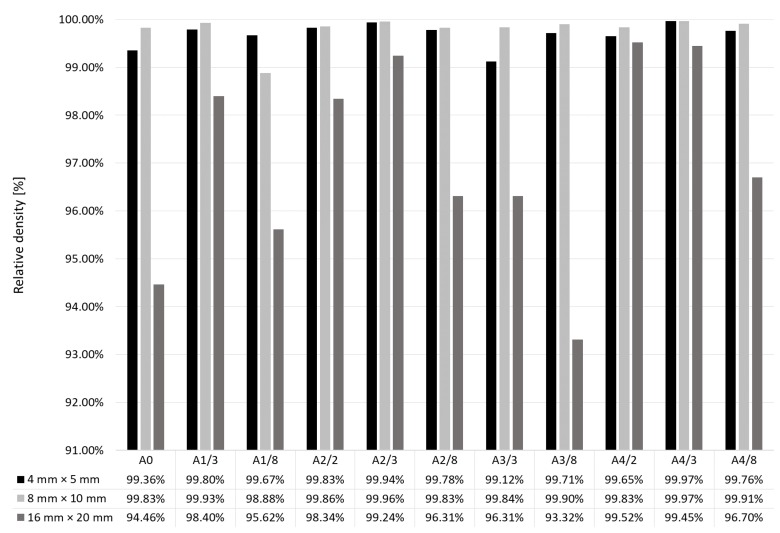
Relative density of specimens manufactured by single scanning with various strategies for various specimen dimensions x × y.

**Figure 7:**
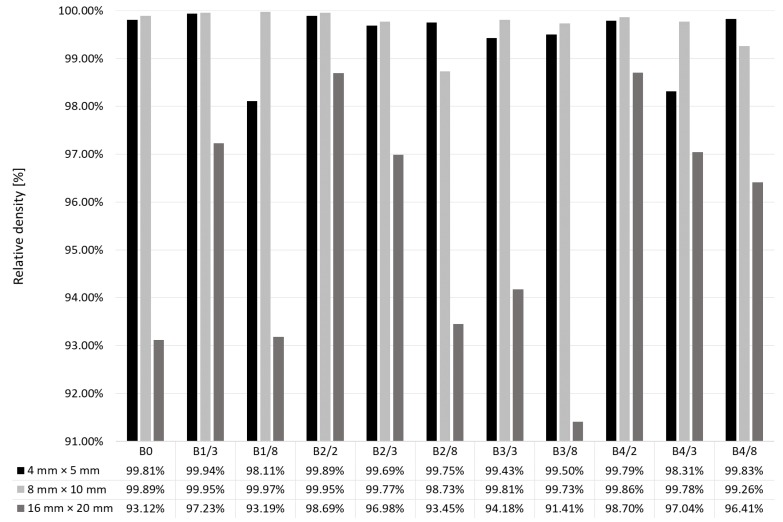
Relative density of specimens manufactured by double scanning with various strategies for various specimen dimensions x × y.

For the research, 22 sets of 3 specimens each were prepared. Results are shown in Figure 6 and Figure 7 for single and double scanning, respectively. It can be seen that, in both cases, the influence of the dimensions of the scanned surfaces xy and scanning strategies on the relative density of the specimens is significant.

All variants of specimens 16 mm × 20 mm did not reach the assumed relative density ≥99.8%. This condition was met by all the specimens (8 mm × 10 mm) produced by single scanning irrespective of strategy, and by the specimens produced by single scanning with the strategies A1, A2 and A4, with the dimension (stripe width or field side length) of the scanned field equal to 3 mm.

Double-scanned small specimens (4 mm × 5 mm and 8 mm × 10 mm) did not result in higher relative density, but the influence of scanning strategy was reduced. Nevertheless, similar to single-scanned specimens, a relative density ≥99.8% was reached by those manufactured with the strategies B0, B1, B2 and B4, with no clear relationship with dimensions (2, 3 or 8 mm) of scanning fields.

Summarizing the above results, it can be said that, irrespective of the dimensions of the scanned surface xy, relatively higher density was obtained by single scanning of small individual fields with the chessboard strategy (A4).

**Figure 8:**
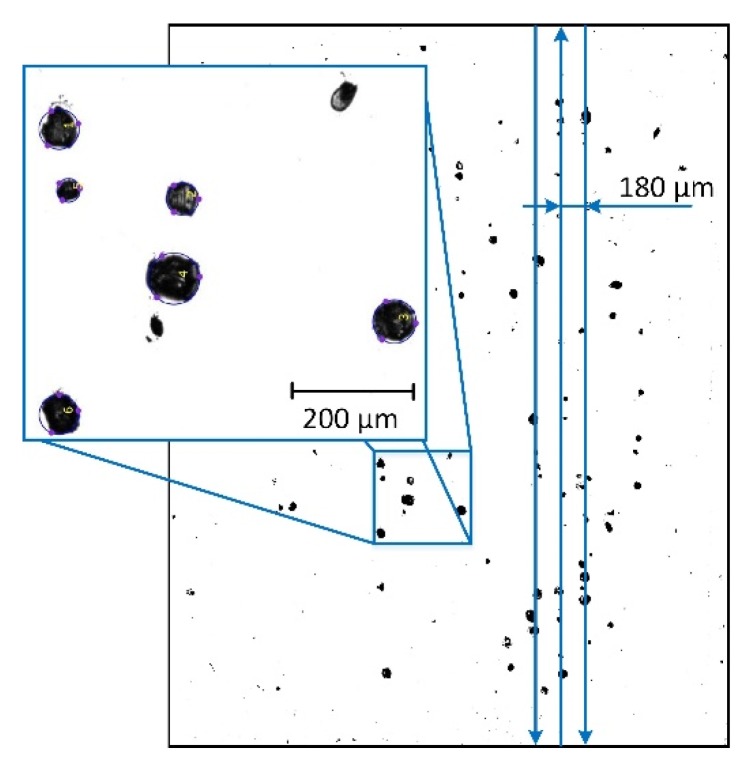
Distribution of pores in specimens manufactured by double scanning with various strategies for various specimen dimensions x × y.

Microscopic examination of the specimens with surface dimensions x × y = 4 mm × 5 mm and 16 mm × 20 mm showed differences in geometry and arrangement of pores. In the specimens 4 mm ×5 mm, pores were spherical with diameter of 20 to 40 µm, linearly arranged along the scanning direction (Figure 8). Similar pores were also observed in both specimens 8 mm ×10 mm and 16 mm ×20 mm, but their amount was significantly smaller than that of pores with different shapes.

**Figure 9:**
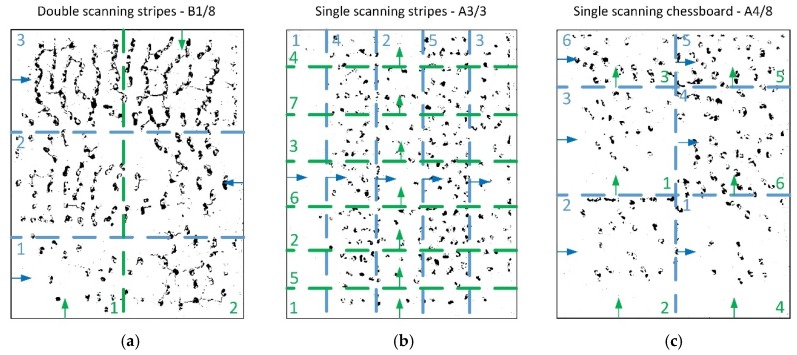
Distribution of pores (black) in the plane xy for the specimens 16 mm × 20 mm produced with scanning strategy: (**a**) B1/8; (**b**) A3/3; (**c**) A4/8. Scanning direction and sequence for even layers (x—green) and odd (y—blue) are presented. During double scanning, the specimen is scanned with the strategy for both even and odd layers.

In the specimens 16 mm × 20 mm, pores in the plane xy were mostly elliptical, with substitute diameter from 150 to 1400 µm. A smaller number of pores was visible in the initial scanning areas, and their concentration increased with proceeding scanning of subsequent sections. Figure 9a shows that the pores can merge to create longitudinal tunnels in the plane xy. No similar effects were observed in the specimens 4 mm × 5 mm and 8 mm ×10 mm.

**Figure 10:**
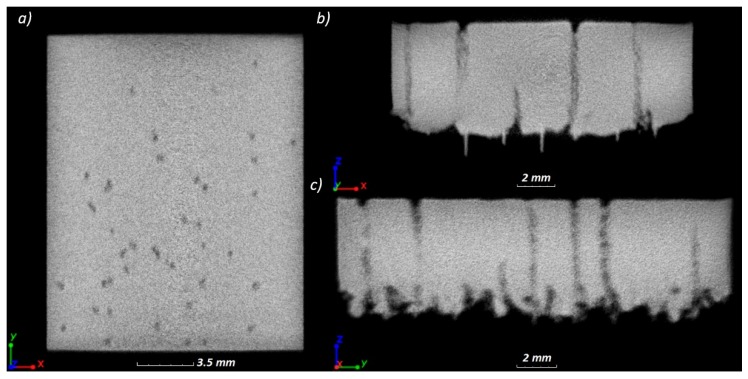
CT image of the specimen A4/3 with marked tunnel-like pores: (**a**) plane xy; (**b**) plane xz; (**c**) plane yz.

**Figure 11:**
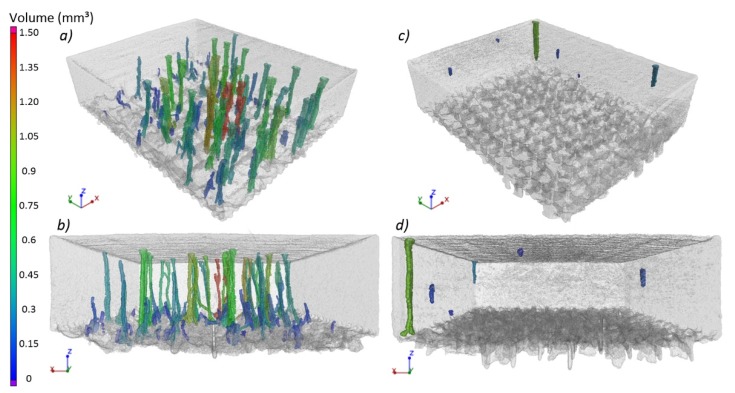
3D visualization of CT examination of the specimens produced: (**a,b**) with the use of supporting structures 2 mm and the strategy A4/3; (**c,d**) with the use of supporting structures 1.5 mm/5° and the strategy A4/2.

The result of CT analysis of the specimen 16 mm ×20 mm made with the strategy A4/3, in the form of reconstruction, is shown in Figure 10. It can be seen that the pores shown in Figure 9a have shapes of columns passing through the entire height of the specimen. Visualization 3D of these pores is shown in Figure 11a,b. It was also found that columnar pores occur in the entire volume of the examined specimen. The tunnel-like shape of pores is the cause of the significant difference between results of quantitative 3D analysis of porosity performed by CT, i.e., 1.51%, and of quantitative 2D analysis performed on xy sections of the specimens, i.e., 0.55%.

**Figure 12:**
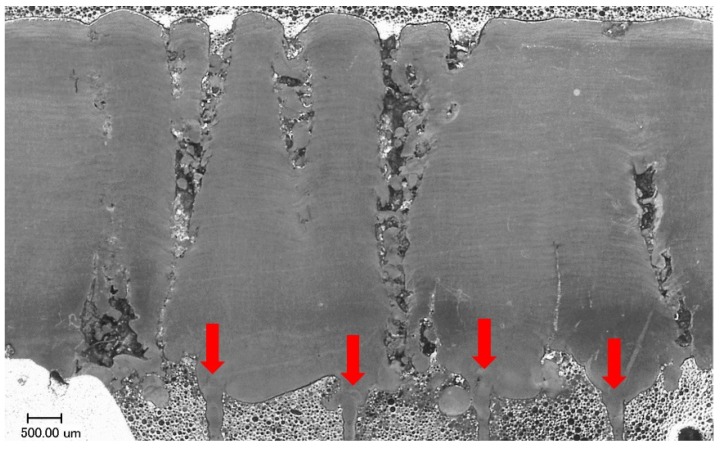
Columnar pores on section xy of the specimen A2/4. These pores are formed between supports shown with red arrows.

**Figure 13:**
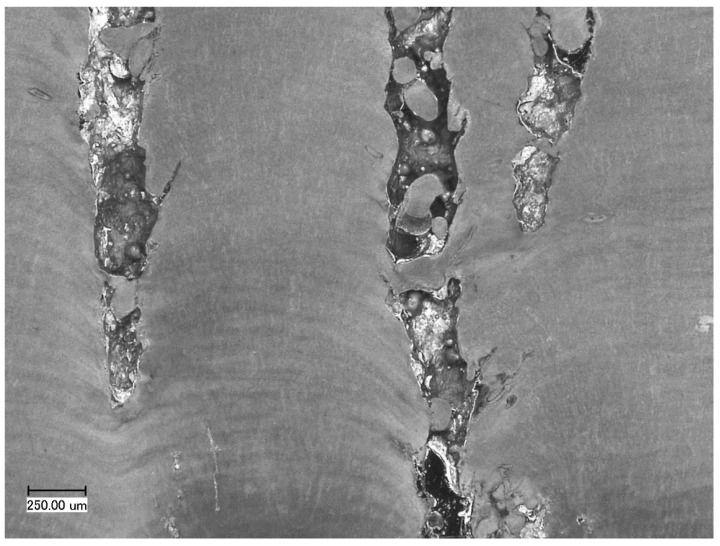
Columnar pores on section xz of the specimen A2/4. Visible sinking of the layer, preceding initiation of pores, and non-melted particles inside pores.

It is visible in Figure 11 that a part of columnar pores (dark blue) disappears with scanning of subsequent layers. Moreover, columnar pores can split and develop in the form of two smaller columnar pores (Figure 12). In the upper part of the specimen, columnar pores expand to create characteristic funnels. Inside the pores, partially re-melted powder particles can be observed, as well as the balling effect on the free surface of solidified material (Figure 13). Due to their character, columnar pores are similar to the lack of fusion type pores. It can be seen in Figure 12 and Figure 13 that columnar pores are created between supporting structures and clear “sinking” of the solidified layers evidences “pouring” of selectively melted metal into the pores.

**Figure 14:**
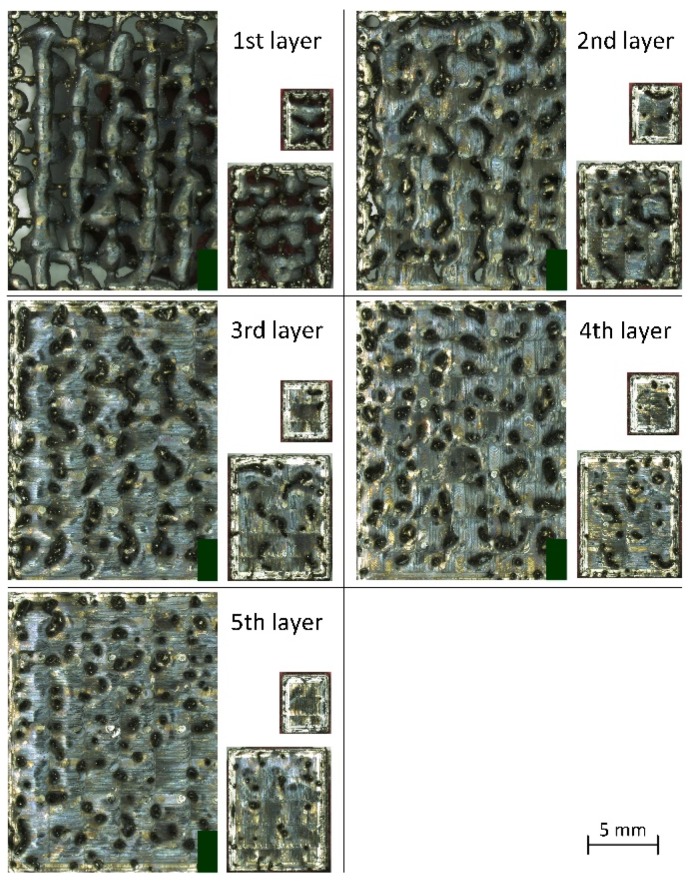
Microscopic images of free surface xy of the specimens with 3 sizes built to the height of 1 to 5 layers with the strategy A4/2.

**Figure 15:**
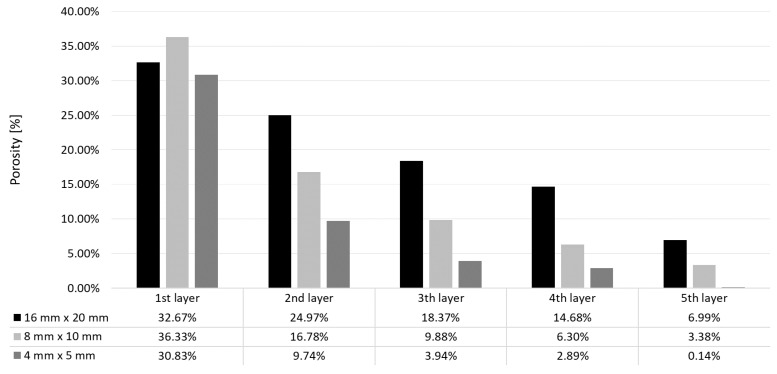
Porosity of individual layers for the specimens A4/2, with dimensions x × y = 16 mm × 20 mm, 8 mm × 10 mm and 4 mm × 5 mm.

For more precise observation of the phenomenon of disappearance of pores, a series of flat specimens 4 mm × 5 mm, 8 mm × 10 mm and 16 mm × 20 mm were manufactured, the height of each corresponding to 1 to 5 layers. Images of xy surfaces of the specimens made with the scanning strategy A4/2 are shown in Figure 14. It can be seen that, for the specimens 4 mm × 5 mm, pores disappear after 5 layers. For the specimens 8 mm ×10 mm, after scanning 5 layers, columnar pores are still visible with maximum dimension up to ca. 800 µm. However, the fifth layer of the specimens 16 mm × 20 mm is characterized by numerous pores that often merge to create linear discontinuities with a circular cross-section. Measurements of percentage of these pores (open) for all the specimens are shown in Figure 15.

As can be seen from Figure 15, porosity decreases with the manufacture of subsequent layers. The specimen x × y = 8 mm × 10 mm is characterized by the highest open porosity for the first layer However, with the manufacture of subsequent layers, the highest porosity occurs in the specimens 16 mm × 20 mm. The largest drops of porosity with increasing number of layers were noted for the specimens 4 mm × 5 mm, and the smallest drops—for the specimens 16 mm × 20 mm.

### 3.2. Effect of Support Structures

Supporting structures are mostly used in order to reduce deformation of the built object. In addition, their task is to remove heat and to support the object. The minimum distance between support structures should be such that the solidifying layers do not sink. However, concentration of supports results in a longer manufacturing time, as well as more difficult removal of the structures and detachment of the model from the working platform.

**Figure 16:**
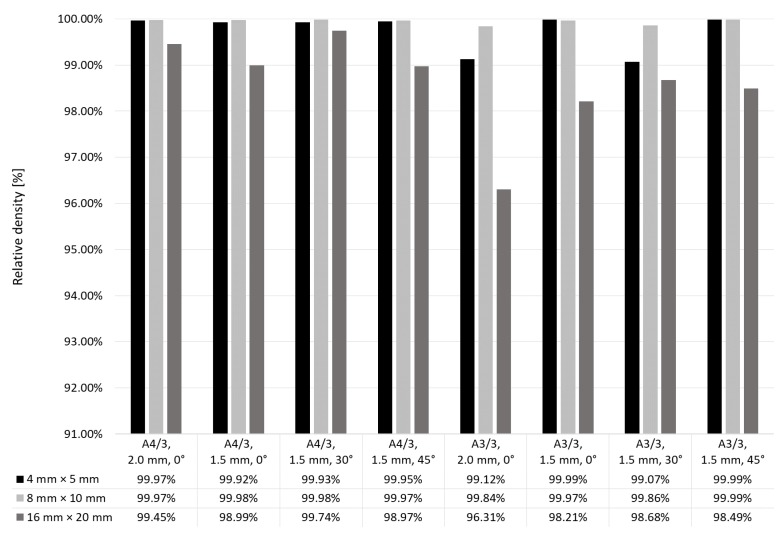
Relative density of specimens depending on their scanning and support strategies.

Considering the fact that columnar pores in Figure 12 and Figure 13 were created between supporting struts, new support structures were used in stage II, with the distance reduced from 2 mm (stage I) to 1.5 mm, with various inclination (0°, 30° and 45°) to the working platform and two scanning strategies: A4/3 and A3/3. These strategies were selected because of very high (A4/3) and very low (A3/3) relative density obtained with their use. Results are shown in Figure 16. Manufacture of the specimens 16 mm × 20 mm with the use of concentrated supporting structures inclined at 30° to the scanning direction resulted in higher relative density, irrespective of scanning strategy. Relative density was improved by 0.29% only for the strategy A4/3 and as much as by 2.37% for the strategy A3/3.

### 3.3. Effect of Intermediate Layers

Supporting structures are unavoidable in SLM processes; nevertheless, the efforts aimed at minimizing their side effects (increased printing time, cost and impact on surface quality) are necessary. In turn, a restriction for minimization of supporting structures is increasing porosity generated during re-melting of the first layers connected with the supports. According to the authors [20], manufacture (at properly selected laser power) of 20 intermediate layers between the minimized supporting structure and standard layers of the built object prevents an increase of porosity and formation of columnar pores.

Thus, at this stage of the examinations, two series of specimens were manufactured:

- at laser power reduced from 200 W to 150 W in order to decrease the tendency of the first solidifying metal layers to overhang,
- at the same laser power of 200 W but with introduced an intermediate layer, i.e., the first five layers built at lower laser power of 125 W (to decrease volume energy density in order to obtain a stable pool of liquid metal).

**Figure 17:**
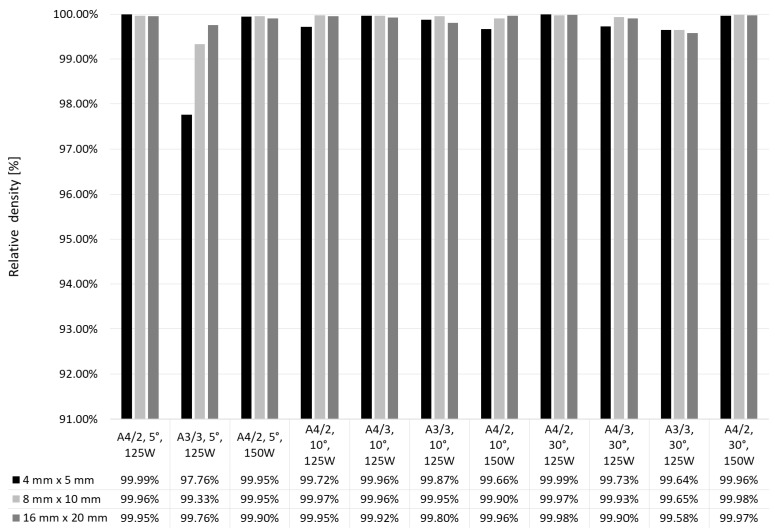
Effect of scanning parameters of intermediate layers and support geometry on relative density.

Specimens were manufactured on supports with distances 1.5 mm, inclined to the axis X at 5°, 10° and 30°. As can be seen in Figure 17, use of the intermediate layer built at lower laser power influenced relative density of the specimens. In comparison to the specimens with no intermediate layers, with inclination angle of supporting structures equal to 30° and with the scanning strategy A4/3, relative density of the specimens 16 mm × 20 mm increased by 0.16%. For the strategy A3/3, relative density increased by 0.90%. For most of the specimens manufactured at stage III, obtained was relative density over 99.5%. Only one parameter set, i.e. laser power 125 W; strategy A3/3 and supports inclined at 5° for manufacture of specimens 4 mm × 5 mm and 8 mm × 10 mm, did not give such satisfactory results. Very low porosity below 0.1% was obtained by the specimens made at laser power 150 W with supporting structures inclined at 5° and 30°.

## 4. Discussion

In the SLM technology, both constant and variable process parameters influence the quality of manufactured parts. Constant parameters should be fixed in a strictly determined time interval. Variable parameters are controlled by an operator in such a way to ensure a stable run of the process and possibly high quality of the manufactured part. A large number of variable parameters makes it impossible to determine the influence of their possible combinations on product quality. Thus, efforts are made to find such parameters that would consider the total effect of the most important variable parameters. They include laser energy density defined by various formulas given in Table 3. The most often used comparable parameter is volume energy density (VED_H_), whose definition considers mostly main variable parameters:

- laser power (W)—P,
- layer thickness (µm)—L,
- hatch spacing (µm)—H,
- scanning speed (mm/s)—V,
- focused beam diameter (µm)—f.

The choice of the definition of energy density depends on the geometry of the manufactured object. For the objects in the form of thin walls, spatial structures and supporting structures, surface energy density is used as a parameter (Equations (2) and (3) in Table 3). In the case of solid parts, the parameter considering hatch spacing is volume energy density (Equations (4) and (5) in Table 3). Most often, the comparative parameter VED_H_ is applied, since it considers the variable parameters most strongly influencing the efficiency of the melting process. However, this is not an ideal parameter, since it does not consider complex physical phenomena, like thermocapillary convection, hydrodynamic instability and recoil pressure that influence transfer of mass and heat inside the molten metal pool [21,22]. Therefore, for comparative reasons, process windows for the manufacture of parts from H13 powder ensure relative density over 99.9%. Table 4 includes values of energy density calculated according to the formulas cited in Table 3.

It can be seen from Table 4 that high density of H13 specimens can be obtained within a wide range of heat input, defined as the ratio of laser power to scanning speed, i.e., 0.25–2.0. High input energy, much higher than that applied by the authors in References [23,24], did not cause the balling effect or any significant increase of surface roughness, similar to results from References [25,26]. In spite of high heat input, which could result in increased width of seams, obtaining high relative density in this research did not require larger values of hatch spacing.

Even though the volumetric energy density parameters VED_H_ and VED_f_ consider differences in focused beam diameter, layer thickness and hatch spacing, they cannot be comparative parameters or serve as design parameters in the optimization process. The obtained results indicate that, apart from heat input, selection of scanning strategy and support strategy play important roles in striving for maximum relative density.

The tendency of decreasing density along with increasing dimensions of scanning surfaces, observed in our own research, should be considered in drawing conclusions from a comparison of SLM parameters, like that in Table 4. The specimens manufactured in [24] had smaller dimensions, which reduced the probability of occurrence of tunnel-like pores.

The result of a comparison is also influenced by the method of porosity measurements. Computed tomography makes it possible to determine the quantity, size and arrangement of pores in the entire volume of the specimen. However, for materials with high density, to which H13 steel belongs, limited resolution of laboratory CT restricts detection of small pores. Resolution of the CT can be increased by reducing the thickness of samples (e.g., by cutting out their fragments) or using a system with higher X-ray tube voltage and current. In Reference [24], resolution of measurements is not quoted. In Reference [23], like in the presented work, relative density measurements were analyzed with the use of image analysis of similar cross-sectional areas, i.e., 100 mm^2^ and 80 mm^2^. No columnar pores occurred in both cases.

Until now, few works have been published in which the occurrence of columnar pores was observed in metal alloys processed by SLM. In one of them, Bauereiß et al. [15], in their experimental work supported by numerical simulation, showed that columnar, channel-like pores in Ti6Al4V occurred in the case when the SLM process was operated at a too low energy density. They also found that, at constant scanning speed and layer thickness, the number and size of channel-like pores decrease with increasing laser power. In this work, however, the opposite tendency of disappearing columnar pores at lower laser power was observed. Nevertheless, it should be stressed that the channel-like defects observed in [15] differed more with more irregular shapes of cross-sections in comparison to more compact, rounded shapes of cross-sections of columnar pores shown in Figure 11 to Figure 13. This can result from various mechanisms of their origin under the conditions of too low energy density (lack of fusion pores) or too high energy density (keyhole induced pores).

Shapes of cross-sections of columnar pores in Figure 10 to Figure 13 are undoubtedly related to geometry of the designed supporting structures and physical factors like wettability, surface tension or gravitation. It can be seen that metal solidifying on the supports tends toward the spherical shape in order to minimize its surface energy. The places most exposed to the occurrence of pores are, first of all, spaces between subsequent supports, as well as between supports and outside edge of the specimen. According to Reference [27,28], geometry and continuity of individual tracks in a single layer are affected by many factors, among others, packing density of powder, scanning speed and ratio of laser power to scanning speed. This applies to building tracks in free powder. When applied, are supports on that solidifying metal “anchors”, seams “develop” along with scanning of subsequent layers and liquid metal spreads flowing to the spaces filled with powder. This is why, in microscopic images (Figure 13), single powder particles or splashes inside columnar pores can be observed. Therefore, in the light of the performed research, it seems purposeful to produce a properly selected number of the first densest layers at reduced laser power, in order to maximize density of the entire manufactured part.

## 5. Conclusions

In this study, the SLM process parameters optimized for H13 steel in relation to the assumed density ≥ 99.8% were used to produce specimens with various dimensions of scanning planes, as well as various scanning and support strategies. The following conclusions can be drawn:

- Application of the same process parameters in the manufacture of specimens with various scanning surface areas results in a decrease of relative density for larger sizes to the degree dependent on the applied scanning strategy.
- Scanning strategy significantly influences quantity, sizes and arrangement of pores. Proper selection of strategy (e.g., chessboard with small dimensions of individual fields) makes it possible to suppress influence of size of the scanning section on relative density of the manufactured parts.
- Double scanning does not significantly reduce porosity of specimens, but reduces the influence of the scanning strategy.
- Distances between supports and their inclination angle to the X axis influence the occurrence of columnar pores. Density of supports and their proper inclination make it possible to reduce the number of columnar pores.
- Application of intermediate layers (first layers from sides of supports, made at properly selected process parameters) makes it possible to reduce the quantity of columnar pores and to maximize density in the entire specimen volume.

## Figures and Tables

**Table 1:** Chemical composition of H13 powder.

Concentration (wt %)	Fe	Cr	Mo	Si	V	Mn	C	P	S
AISI H13	Reminder	4.75–5.50	1.10–1.75	0.8–1.25	0.8–1.20	0.2–0.6	0.32–0.45	0.03	0.03
Used powder	Reminder	5.07	1.72	0.88	1.02	0.44	0.41	0.008	0.009

**Table 2:** Process parameters used for the manufacture of all the specimens.

Laser Power (W)	Exposure Time (µs)	Distance between Scanning Points (µm)	Hatch Distance (µm)	Layer Thickness (µm)	Scanning Speed (mm/s)
200	800	80	180	50	100

**Table 3:** Definitions of energy density used in powder-bed fusion additive manufacturing.

Item	Designation	Definition	Unit	Reference
1	Surface power density	$P_{s}=\frac{4P}{\pi f^{2}}$	$\frac{W}{mm^{2}}$	-
2	Surface energy density	$E_{l}=\frac{P}{V\cdot L}$	$\frac{J}{mm^{2}}$	[13]
3	Surface energy density	$E_{f}=\frac{P}{V\cdot f}$	$\frac{J}{mm^{2}}$	-
4	Volume energy density	$VED_{H}=\frac{P}{V\cdot H\cdot L}$	$\frac{J}{mm^{3}}$	[13]
5	Volume energy density	$VED_{f}=\frac{P}{V\cdot f\cdot L}$	$\frac{J}{mm^{3}}$	-

**Table 4:** Comparison of process parameters and corresponding energy densities used for the manufacture of H13 specimens with relative density >99.8%.

Parameter	This Work A0	This Work A4/3	This Work A4/2, 1.5 mm, 30°, 150 W	Laakso [23]	Mazur VED 80 [24]	Mazur VED 120 [24]
Laser power (W)	200	200	150	251	225	375
Layer thickness (µm)	50	50	50	30	30	30
Scanning speed (mm/s)	100	100	100	994	781	868
Hatch spacing (µm)	180	180	180	100	120	120
Focused beam diameter (µm)	200	200	200	80	80	80
P_s_ (W/mm^2^)	6369	6369	4777	49863	44785	74642
E_l_ (J/mm^2^)	40	40	30	8	10	14
E_f_ (J/mm^2^)	10	10	7.5	3.15	3.6	5.4
VED_H_ (J/mm^3^)	222	222	167	84	80	120
VED_f_ (J/mm^3^)	200	200	150	105,04	120	180
Strategy	A0	A4/3	A4/2	Rotated 67°	N/A	N/A
Specimen dimensions (mm^3^)	8 × 10 × 5	8 × 10 × 5	8 × 10 × 5	10 × 10 × 10	4 × 4 × 4	4 × 4 × 4
Method of relative density measurement	Image analysis without boundary	Image analysis without boundary	Image analysis without boundary	Image analysis without boundary	CT	CT
Relative density (%)	99.83	99.97	99.98	99.91	99.91	99.99
